# Old and new strategies in therapy and diagnosis against fungal infections

**DOI:** 10.1007/s00253-023-12884-8

**Published:** 2024-01-19

**Authors:** Tania Vanzolini, Mauro Magnani

**Affiliations:** Department of Biomolecular Sciences, University of Urbino Carlo Bo, 61029 Urbino, PU Italy

**Keywords:** Fungi, Antifungal drugs, Antifungal treatment, Antifungal diagnostics, Resistance, Antifungal target

## Abstract

**Abstract:**

Fungal infections represent a serious global health threat. The new emerging pathogens and the spread of different forms of resistance are now hardly challenging the tools available in therapy and diagnostics. With the commonly used diagnoses, fungal identification is often slow and inaccurate, and, on the other hand, some drugs currently used as treatments are significantly affected by the decrease in susceptibility. Herein, the antifungal arsenal is critically summarized. Besides describing the old approaches and their mechanisms, advantages, and limitations, the focus is dedicated to innovative strategies which are designed, identified, and developed to take advantage of the discrepancies between fungal and host cells. Relevant pathways and their role in survival and virulence are discussed as their suitability as sources of antifungal targets. In a similar way, molecules with antifungal activity are reported as potential agents/precursors of the next generation of antimycotics. Particular attention was devoted to biotechnological entities, to their novelty and reliability, to drug repurposing and restoration, and to combinatorial applications yielding significant improvements in efficacy.

**Key points:**

*• New antifungal agents and targets are needed to limit fungal morbidity and mortality.*

*• Therapeutics and diagnostics suffer of delays in innovation and lack of targets.*

*• Biologics, drug repurposing and combinations are the future of antifungal treatments.*

## A newly found old threat

Even if part of the microbiological world, fungi have never had the consideration that bacteria, and viruses on the contrary received. The underestimation of their risk led to a minor interest from the scientific community hence, to less information and drugs currently available. Fungi share with their hosts many similarities proper of eukaryotic organisms, making the hunt for effective and safe antifungal drugs incredibly challenging from both the scientific and economic point of view. Moreover, several bad behaviors and practices have worsened the situation like the extensive and intensive use of antifungal agents in agriculture, or the incorrect use by patients. In response to our negligence, now we must face not just the great plague of fungal infections itself, but also the rise, reappearance, and spread of species carrying intrinsic and multidrug resistance. This review considers a well-known threat that is quickly becoming more dangerous. Our aim is not just giving a concise description of the state-of-the-art but offering a real insight toward the next generation of antifungal entities. We examined old and new treatments of natural, semi-/synthetic origin allowing great consideration to biotechnological agents. With a fresh point of view, we tried to find logical interrelationships among pathways and, therefore, among drug activity that affect different and interconnected players. We showed the options available and under investigation with extremely updated information and we condensed (when relevant and known) details that in previous works were only superficially or partially discussed like characteristics, mechanisms of action, spectra, pharmacokinetics, toxicities, positive and negative interactions, and, about that, combinatorial approaches and repurposing strategies. Finally, we also faced a hot topic represented by diagnoses that are affected by poor novelty, slowness, and inaccuracy—aspects that fall back into delayed therapies and negative outcomes in terms of patient survival.

## Diagnosis

Diagnosis is an important issue, especially for fungal infections. Despite the development of new diagnostic agents and techniques, fungal identification relies on (from the most to the least used) microscope examination, microbial cultures, antigen detections, serological tests, and molecular analyses (Kozel and Wickes 2014). The first two processes are based on the growth of fungi in plates or liquid medium and are followed by susceptibility testing to find MICs and breakpoints for the correct use of antifungal drugs. Despite these procedures being still largely employed, they delay the beginning of the treatment and negatively affect patient recovery or survival. Moreover, the accuracy is lower compared to more recent techniques as molecular and immunological assays. However, about them, less information is available on online platforms, and databases are poor and limited to a few and more common mycoses (Zhang et al. 2021b). The presence in the market of diagnostic kits for antigen detection is also restricted, and sensitivity and specificity are not always optimal (Wickes and Wiederhold 2018; Rodrigues and Nosanchuk 2020). Finally, there is patient variability: symptoms are diversified from subject to subject even if the infection is caused by the same pathogen. In this regard, the degree of infection, comorbidity, and physio-pathological and pharmacological conditions like immunodeficiency or immunosuppressive treatments are associated with the fastest progressions and worst outcomes. This sad view highlights the urgent need for not just new antifungal drugs but also for new data and techniques for clear, easy, quick-to-perform, robust, sensitive, and specific diagnoses and diagnostic tools.

## Therapy

### The old

The battle against bacteria continually met fast times of new target identifications and compound developments. Conversely, the hunt for antifungal drugs was characterized by long and dilated times for effective innovation; hence, the fungal plague was always combated with a poor arsenal and limited suitable targets. Only in recent years have fungal infections been figured out as one of the most serious health problems of our time and for the near future, but the risks that they represent are not even remotely comparable to the number of available therapeutic options. Currently, a few classes of drugs are used in clinics and veterinary.

#### Polyenes

Amphotericin B is the major representative of the polyene class. It is a fungicide able to interfere with the ion homeostasis of the fungal cell, establishing hydrophobic interactions with ergosterol of the membrane and creating pores. It also induces an accumulation of reactive oxygen species and both mechanisms contribute to cell death. Since its discovery in 1955, more than 200 polyenes have been developed, but amphotericin B keeps being the drug of choice especially for systemic mycoses (Houšť et al. 2020). Although its high efficiency, its main limitations are represented by nephro- and hepatotoxicity, infusion reactions, and electrolyte anomalies (Laniado-Laborín and Cabrales-Vargas 2009; Nett and Andes 2016); therefore, from the deoxycholate form, new formulations were elaborated in order to ameliorate both safety and effectiveness. The most well-known are the lipid-based formulations that consist of the drug loading into liposomes or lipid complex, a colloidal dispersion and microemulsions (Marena et al. 2022).

#### Azoles

After their discovery, rapid azoles became the first-line drugs. Apart from a few exceptions, they are considered organism-dependent fungistatic/fungicidal (Manavathu et al. 1998) and can inhibit the cytochrome P450-dependent lanosterol-14α-demethylase, an essential enzyme in the synthesis of ergosterol (Mast et al. 2013). This class contains both imidazole- and triazole-active group molecules since several modifications in the structure followed one another, and gave birth to different generations. First generation is occupied for instance by clotrimazole, ketoconazole, miconazole, butoconazole, econazole, bifonazole, terconazole, tioconazole, etc., which present the imidazole ring. The scarce water solubility was an obstacle as well as the limited oral bioavailability and side effects like gastrointestinal and endocrine alterations and hepatotoxicity. Even if their uses decreased over time, especially for invasive infections, they are still adopted in topical preparations. Second and third generations exhibit a triazole ring in the formula and underwent structural changes that progressively resulted in increased efficacy and hydrosolubility, extended spectra, better pharmacokinetics and pharmacodynamics, lower toxicity, and new possible formulations. Fluconazole and itraconazole stand in the second generation while voriconazole, posaconazole, isavuconazole, ravuconazole, and albaconazole in the third (Nett and Andes 2016). They are widely used as treatment, prophylactic, and pre-emptive both in medical and surgical fields (Allen et al. 2015). Despite them being well-tolerated, a few restrictions have been highlighted like the QT prolongation, the teratogenicity, and the drug–drug interactions of triazoles (Pursley et al. 1996; Nett and Andes 2016; Cook et al. 2017).

#### Echinocandins

Echinocandins represent one of the most recent classes of antifungal drugs starting its development in the early 2000s. They are semisynthetic lipopeptides with a structure resembling fermentation metabolites of different microorganisms (Houšť et al. 2020). Echinocandins inhibit in a non-competitive manner the UDP-glucose-β-1,3-D-glucan-β-3-D-glucosyltransferase also known as β-1,3-D-glucan synthase, an essential enzyme for the synthesis of β-1,3-glucans, fundamental components of the fungal cell wall. β-1,3-D-glucan synthase is a complex formed by two subunits, one regulatory, Rho1 (Ras homologous 1), and one catalytic, Fks (FK506 **s**ensitivity); in particular, Fks is encoded by three genes, FKS1, FKS2, and FKS3. Echinocandins seem to target specifically the FKS1 gene (Loh and Ang 2020). Based on the fungus and also on the species and strain, echinocandins can be considered either fungistatic or fungicidal. Caspofungin, micafungin, and anidulafungin were the most successful compounds that reached the market. Their strong activity associated with a broad spectrum, good pharmacokinetics and pharmacodynamics, high safety, and few drug–drug interactions makes them suitable as first-line drugs, especially in nosocomial candidemia (Kato et al. 2021).

#### Pyrimidine analogs

5-Flucytosine is the only component of this class. From its approval in the 1960s, it was considered for a long time just an antimetabolite for cancer treatment, but its low antineoplastic activity pushed it toward the antimicrobial pipeline as an antifungal drug. 5-Flucytosine is a fluorinated analog of cytosine, and for this reason, it can easily enter the fungal cells through the cytosine permease and be deaminated by cytosine deaminase to 5-fluorouracil. Then 5-fluorouracil is converted into 5-fluorouridine triphosphate able to be incorporated in fungal RNA, hence, altering the aminoacylation of tRNA and inhibiting the protein synthesis. At the same time, 5-fluorouracil, when metabolized into 5-fluorodeoxyuridine monophosphate, inhibits the thymidylate synthase, an essential enzyme for DNA synthesis and nuclear division (Houšť et al. 2020). This double mechanism of action makes 5-flucytosine a potent drug, especially in candidiasis and cryptococcosis, but it can be used only in combination with other drugs like amphotericin B because of the rapid development of resistance (Chandra et al. 2009). It has high oral bioavailability but a brief half-life. The interactions with other drugs are few but the main limitations are referred to bone marrow suppression and hepatotoxicity (Nett and Andes 2016).

#### Allylamines

Allylamines represent a small family of molecules that target the squalene epoxidase (or squalene monooxygenase) an enzyme needed for the ergosterol synthesis (Mota Fernandes et al. 2021). They are not commonly used in clinics except for dermatophytes against whom they are fungicidal (Ward et al. 2022). Terbinafine, naftifine and also pentamidine, and some derivatives like butenafine or amorolfine are examples of squalene epoxidase inhibitors.

### The new

Some new strategies were found in recent years. As safety is the most important issue in patient treatment, the reasonable logic for target identification relies on the differences between fungal and host cells. Herein are reported molecules with antifungal properties acting on pathways presenting significant discrepancies between fungal and mammalian cells.

#### Hsp90-calcineurin-PKC axis

**Fig. 1:**
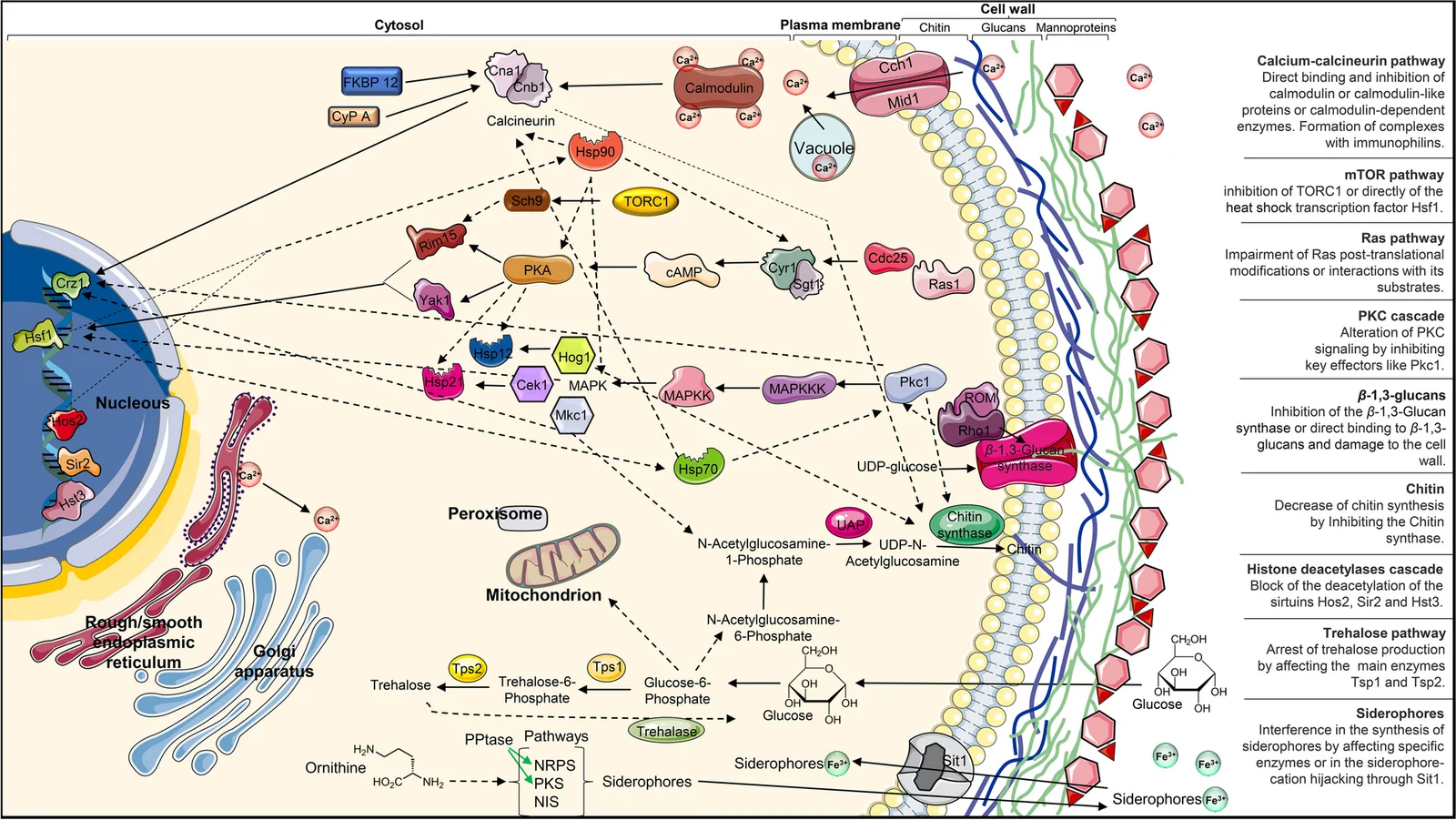
Overview of the structural organization of a model fungal cell with a focus on the mechanism and pathway sources of potential antifungal targets. The pathways reported are those occurring in the cytosol or linked to the plasma membrane and cell wall. Arrows show the deep and complex interconnection among pathways giving a sight of the consequences of the activity of a single drug among the interconnected players and a possible explanation of the interactions between drugs

Among the relatively new targets, it is important to mention the calcineurin pathway (Fig. 1). Calcineurin is a Ca^2+^/calmodulin-activated protein phosphatase 2B essential for activating stress responses inside the host, but it also plays a role in fungal growth, morphotype change, virulence, and resistance development (Zheng et al. 2018). In response to stimulation, the calcium channel CMC (**C**ch1-**M**id1 **c**hannel) in the plasma membrane lets Ca^2+^ ions enter the cell and bind calmodulin. The activated Ca^2+^-calmodulin complex, in turn, binds the **c**alcineurin (Cn) heterodimer (CnA and CnB) which can dephosphorylate the calcineurin-dependent transcription factor **c**alcineurin-responsive zinc finger 1 (Crz1). Crz1 then translocates into the nucleus and activates the transcription of genes related to stress response, cell wall integrity, growth, and drug resistance. Calcineurin is finely modulated by immunophilins cyclophilin A (CyP A) and FK506-binding protein 12 (FKBP 12). These proteins are the target of immunosuppressors like tacrolimus (FK506) and cyclosporine A, mainly used to avoid rejection after transplantations (Mota Fernandes et al. 2021). They bind immunophilins forming drug-immunophilin inhibitory complexes which impair the interaction of calcineurin to its substrates. Recently they received large interest for their synergistic effect in combination with caspofungin and azoles against different fungal species, echinocandins- and azoles-resistant strains as well, having an inhibitory activity also on the biofilm formation (Uppuluri et al. 2008; Lamoth et al. 2013; Şen Kaya et al. 2021). Nevertheless, because of the serious consequences on the immune systems, different efforts were made to limit side effects while still maintaining the antifungal activity. APX879, the successor of tacrolimus, has high efficacy in in vivo cryptococcosis but low immunosuppression (Juvvadi et al. 2019). New calcineurin inhibitors were discovered. Among them, there are vasostatin-I which is a peptide derived from chromogranin A, its shorter fragments chromofungin and catestatin, which are active against yeasts and filamentous fungi (Aslam et al. 2012; Jati et al. 2023), and tamoxifen, an anticancer that is supposed to interfere in the calcineurin pathway and that synergizes with amphotericin B (Hai et al. 2019).

Calcineurin’s effectiveness is often associated with one of its upstream regulators, the heat **s**hock protein 90 (Hsp90), a chaperon involved in stress signaling pathways, virulence, and resistance (Mota Fernandes et al. 2021). Hsp90 regulates the morphology of fungi differently; its inhibitors induce filamentation in *Candida* spp. (Shapiro et al. 2012) and repress it in *Aspergillus* spp. with negative outcomes in germination and conidiation (Lamoth et al. 2012). Hsp90 modulators, in combination with amphotericin B, azoles, and echinocandins improved the antifungal activity, reduced the mortality rate, and impaired the evolution of resistance (Singh et al. 2009). Mycograb (efungumab) is a monoclonal antibody in a single-chain-fragment variable format. It binds selectively to Hsp90 in the middle region. This inhibits the interaction between the C-terminal and N-terminal regions and prevents the conformational changes needed to perform its activity as a chaperon. Mycograb demonstrated great efficiency in invasive candidiasis (Karwa and Wargo 2009) but never reached the market because of quality and safety issues. Geldanamycin and its derivatives, such as the semi-synthetic variant 17-AAG, are other Hsp90 inhibitors. Besides their antitumoral properties, they showed good antifungal effects. They bind the N-terminal ATP/ADP-binding domain inhibiting the ATPase activity and proved to be effective both alone and in combination. Unfortunately, they also showed hepatotoxicity (Gorska 2012; Liu et al. 2022) mainly related to the cross-inhibition of Hsp90 of the host. For this reason, more selective inhibitors like CMLD013075, are currently under investigation (Whitesell et al. 2019). Other interesting Hsp90-targeting compounds include Ganetespib, able to reverse fluconazole resistance in an in vivo candidemia model (Yuan et al. 2021), and Monorden, suitable for the development of fungicides (Nguyen et al. 2020). Hsp90 is also involved in the maturation and stability of mTOR (mammalian target of rapamycin) effector complexes such as phosphatidylinositol 3-kinase-related protein kinases (PIKKs) (Takai et al. 2010; Millson and Piper 2014). The immunosuppressant rapamycin was demonstrated creating a complex with immunophilin FKBP 12. The complex then binds to the FRB (FKBP12-rapamycin binding) domain, in Tor protein kinases, inhibiting their activity. Tor protein kinases are fundamental components of TORC1 (target of rapamycin complex 1) which regulates a multitude of pathways (especially those nutrient-sensitive). When TOR protein kinases are inhibited by rapamycin, the fungal growth is blocked, and the azole resistance is reversed. Initially, its activity was supposed to be related just to the inhibition of TORC1 that regulates the gene expression of heat shock factor 1 (Hsf1) and, downstream, heat shock proteins like Hsp90 (Deprez et al. 2018). However, recently, rapamycin was observed to exert its antifungal effect also directly on Hsp90 by inhibiting Hsf1 activation without altering TORC1 (Millson and Piper 2014). Its main limitation is the immunosuppressive activity in the host, but some derivatives like everolimus, with reduced drawbacks, are under investigation also in combinatorial treatments (Jiang et al. 2022).

Hsp90 also regulates the signaling of the protein kinase **C** (PKC) cascade (Fig. 1) essential for calcineurin activation, cell wall integrity, growth, morphotype, resistance, and stress responses (LaFayette et al. 2010). Pkc1 is the key kinase of this cascade. It is activated by Rho1 and controls several pathways, including the MAPK (mitogen-activated protein kinase) cascade and the downstream Mkc1 (MAP kinase from *C. albicans*), Cek1 (*C. albicans* ERK-like kinase), and Hog1 (high osmolarity glycerol) signaling. The selective inhibition of Pkc1 by Cercosporamide reduced the virulence in vivo and conferred hypersensitivity to several drugs turning some fungistatic ones into fungicidal ones (Sussman et al. 2004). Moreover, the impaired activation of MAPK-Mkc1 decreased the resistance to ergosterol biosynthesis inhibitors opening the doors to potential upstream agents able to deplete Mkc1 or, in a similar way, Cek1 and Hog1 (Singh et al. 2009; LaFayette et al. 2010). In this regard, magnolol negatively affects *C. albicans* virulence factors by inhibiting PKC and MAPK-Cek1 signaling (Xie et al. 2022). Finally, another option concerns the inhibition of chaperons of the heat-shock protein family which are involved in the cascades. In particular, Hsp90 (see above) directly regulates Mkc1, Cek1, Hog1, and also Hsp70 (for Pkc1), which in turn are both regulated by Hsf1, Hsp21 (for Cek1), and Hsp12 (for Hog1).

At the top of the Hsp90-calcineurin-PKC axis, the histone deacetylases cascade (HDACs, KDACs) (Fig. 1) controls the expression and function of different proteins (Hsp90 included) sometimes involved in fungal virulence. AR-12 is a celecoxib analog protein kinase inhibitor that operates on different targets. It inhibits acetyl fungal coenzyme A synthetase 1 (Acetyl-CoA synthetase 1) causing a downstream repercussion on the metabolism and especially on the histone acetylation. Moreover, it affects Hsp90 and Hsp27 chaperons’ production, downregulating their genes (Rauseo et al. 2020). Noteworthy is Hos2 (HDA one similar), a regulator of Hsp90 deacetylation and activity. Among its inhibitors, there is MGCD290 which is extremely efficient against *Candida* and *Aspergillus* spp., especially when synergically combined with azoles and echinocandins (Rauseo et al. 2020). Trichostatin A is another HDAC inhibitor that enhanced the efficacy of azoles against *C. albicans* and of caspofungin against *Aspergillus* spp. (Bauer et al. 2016). Other interesting enzymes work at this level; when Hst3 is inhibited by nicotinamide, it demonstrates antifungal properties while genetic depletion of acetyltransferases conferred hypersensitivity to fluconazole (Wurtele et al. 2010).

Essentially inhibitors of the Hsp90-calcineurin-PKC axis represent actors that play at different but interconnected layers of the same piece. All the approaches are extremely promising in treating fungal infections.

#### The Ras pathway

Some other pathways are significantly different in fungi and in host cells; therefore, enzymes involved or genes encoding them could be optimal antifungal targets. This is the case of the Ras pathway (Fig. 1) in fungi that is involved in some protein transduction, virulence, morphotype switch, and vital processes (Fortwendel et al. 2004; Hogan and Sundstrom 2009). Ras proteins reach the plasma membrane both in a palmitoylated and not-palmitoylated form. There, some post-translational modifications essential for its biological activity occur, and finally, the interactions with the guanine nucleotide exchange factors (GEFs) like Cdc25 (cell division cycle 25) lead to activation. Brilliant antifungal approaches could exploit the inhibition of palmitoylation or of post-translational modifications such as prenylation. Alternatively, the impairment of the interaction of Ras with GEFs, with its common substrates, or with Ras-related proteins like RhbA are equally valuable strategies (Maurer et al. 2012; Wang et al. 2022). Some farnesyltransferase inhibitors proved to be successful in killing different fungal species (Hast et al. 2011; Qiao et al. 2013). For instance, tipifarnib and lonafarnib reversibly bind to the farnesyltransferase impeding the prenylation of the cysteine residue on the C-terminus of Ras protein (CAAX tetrapeptide motif), preventing its interaction with the plasma membrane and subsequent activation. Other farnesyltransferase inhibitors developed or discovered are farnesyltransferase inhibitor III which is able to block the yeast-hyphal change in *C. albicans* (McGeady et al. 2002), manumycin A, actinoplanic acid A, and its desmethyl analog actinoplanic acid B, isolated from, respectively, *Streptomyces* spp., *Actinoplanes* spp., and both of them (Mrak et al. 2018).

#### Lipids biosynthesis and lipid anchors

**Fig. 2:**
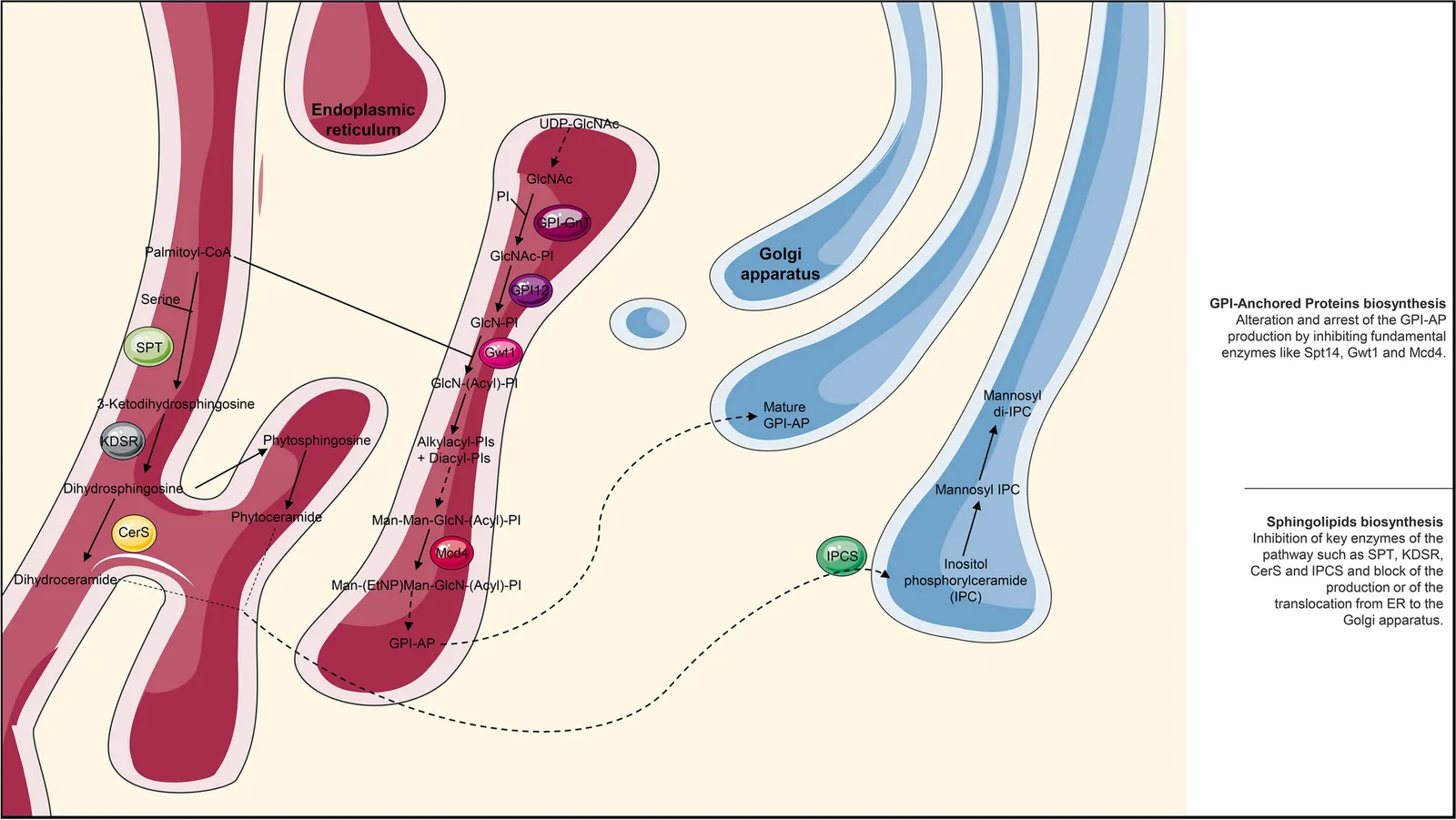
Biosynthetic pathways occurring between the endoplasmic reticulum and the Golgi apparatus which are sources of antifungal targets. Some enzymes are already target of different compounds (Spt14 subunit of the UDP glycosyltransferase which catalyzes the formation of GlcNAc, Gwt1, and Mcd4 for the GPI-anchored protein (GPI AP) biosynthesis and SPT, CerS, and IPCS for the sphingolipids biosynthesis), but others are reported as potential new targets

A similar discourse can be done for sphingolipids biosynthesis (Fig. 2). Even if they are components of the eukaryotic membranes, sphingolipids like inositol phosphoryl ceramide and glucosylceramide are responsible for virulence and drug resistance (Rittershaus et al. 2006; Fernandes et al. 2018). From the top of the sphingolipids biosynthesis, myriocin inhibits **s**erine palmitoyl transferase (SPT), an enzyme that condensates L-serine and palmitoyl-Coenzyme A to produce 3-ketodihydrosphingosine (Yang et al. 2021). 3-ketodihydrosphingosine is reduced through 3-ketodihydrosphingosine reductase (KDSR) in dihydrosphingosine and acylated by ceramide **s**ynthase (CerS). Fumonisin B (Kozel and Wickes 2014) and australifungin act on the ceramide synthase whose impairment makes *C. neoformans* virulent (Delgado et al. 2006). Acylhydrazones like BHBM, D0, and derivatives like D2, D13, D17, and SB-AF-1002 affect the glucosylceramide synthesis by regulating the vesicle sorting or transport between Golgi apparatus and ER (Artunduaga Bonilla et al. 2021). In particular, BHBM and D0 affect the expression of APL5, COS111, MKK1, and STE2 genes which, in fact, are directly involved in vesicular trafficking of sphingolipids and cell cycle progression. Finally, aureobasidin A and its derivatives inhibit the inositol phosphoryl ceramide **s**ynthase (IPCS) which normally leads to the production of inositol phosphoryl ceramide (Aeed et al. 2009). In addition, there are also biological options. A IgG1, IgG2b, and a camelid single-domain antibody (VHH 41D01) showed their affinity for glucosylceramides and demonstrated high efficiency in inhibiting fungal growth. Moreover, the IgG1 offered protection from a lethal dose of *C. neoformans*; the IgG2b enhanced the macrophagic response, and the VHH 41D01 reduced the host inflammation (De Coninck et al. 2017). The recombinant enzyme Cerezyme can hydrolyze fungal glucosylceramides controlling *C. neoformans* infection and having a protective effect (Rhome et al. 2011).

Concerning post-translational modifications, some proteins are supplemented with a glycosylphosphatidylinositol (GPI) group that allows proteins to travel from the endoplasmic reticulum to the plasma membrane and to the cell wall (Fig. 2). An altered biosynthesis of the GPI anchor resulted in defective morphology and attenuated virulence both in *C. albicans* and *A. fumigatus*, meaning that some GPI-anchored proteins have a role in the fungal pathogenesis (Richard et al. 2002; Samalova et al. 2020; Snelders et al. 2022). The GPI-anchor biosynthesis became thereby a source of targets. GPI assembly initiates with the transfer of **N**-acetylglucosamine (GlcNAc) from UDP-GlcNAc to phosphatidyl**i**nositol (PI). This reaction is catalyzed by UDP-glycosyltransferase, and one of its subunits, Spt14 (suppressor of Ty insertion mutations), is the target of Jawsamycin. In the GPI-anchor biosynthesis, inositol acyltransferase Gwt1 (GPI-anchored wall protein transfer 1) mediates the acylation of glucosamine phosphatidylinositol (GlcN-PI) to form GlcN-(Acyl)-PI. Several compounds were found to inhibit Gwt1 like gepinacin, manogepix (APX001A or E1210), and its prodrug fosmanogepix (APX001 or E1211), BIQ, G365, and G884. Another interesting target is the phosphoethanolamine transferase-I Mcd4 (morphogenesis checkpoint dependent 4). Mcd4 transfers an ethanolamine phosphate (EtNP) group to the di-mannosylated GlcN-(Acyl)-PI (Man-Man-GlcN-(Acyl)-PI) to produce Man-(EtNP)-Man-GlcN-(Acyl)-PI. Molecules like YW3548 (M743) and M720 have Mcd4 as targets (Yadav and Khan 2018).

#### The trehalose pathway

One of the most attractive and newest targets is represented by trehalose biosynthesis (Fig. 1). Its absence in mammalian cells dramatically reduces the host toxicity. Trehalose is a storage carbohydrate useful as a carbon source for processes like glycolysis, fungal germination, and sporulation and is an important player in stress conditions. Its pathway involves two main enzymes: the trehalose 6-phosphate synthase 1 (Tps1) and the trehalose 6-phosphate phosphatase (Tps2). The deletion of their genes affected *Cryptococcus* spp., *C. albicans,* and *Aspergillus* spp. growth, survival, and virulence and *Aspergillus* spp. germination. Moreover, a compromised Tps2 causes an accumulation of trehalose 6-phosphate leading to cell death (Thammahong et al. 2017; Chen et al. 2020). Trehalose is used in fungal metabolism and for this reason, it is converted again into glucose thanks to trehalases (trehalose-degrading enzymes) which are α-glucosidase hydrolase. Validamycin A binds competitively with trehalase inhibiting its activity, thereby affecting the glucose metabolism of fungi. Furthermore, Validamycin seems to affect also genes related to MAPK and their ribosome synthesis. Despite these delicious targets, just a few studies have identified effective inhibitors (Yang et al. 2023).

#### The glyoxylate cycle

**Fig. 3:**
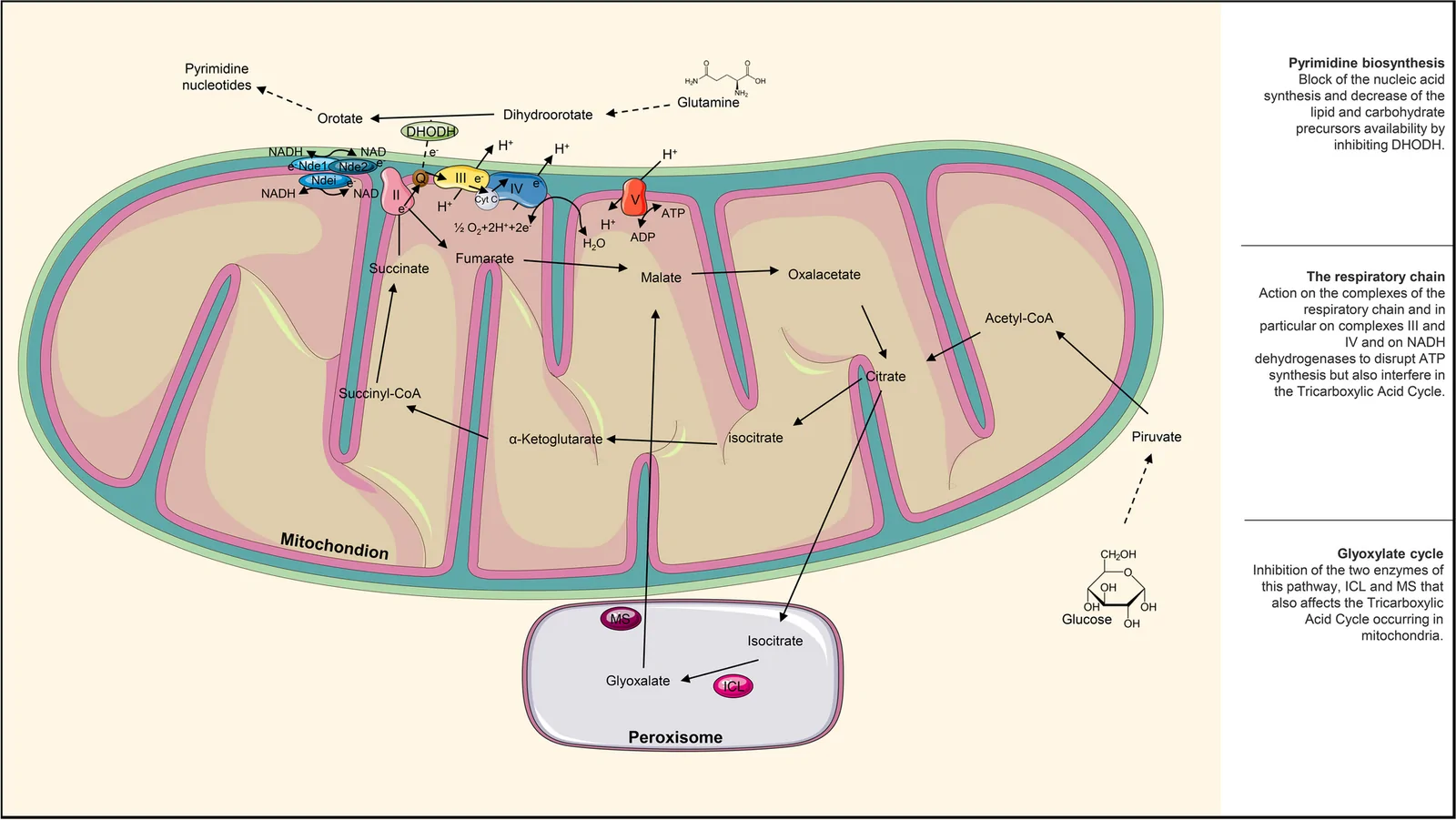
Biosynthetic pathways and metabolic cycles occurring in the mitochondrion (the tricarboxylic acid cycle and the mitochondrial respiration), in the peroxisome (the glyoxylate cycle) or strictly linked to the mitochondrial structures (Pyrimidine biosynthesis). Herein are reported enzymes that are already targeted by different compounds (DHODH, the respiratory complexes, ICL, and MS) and potential new targets

The glyoxylate cycle (Fig. 3) seems to be important for virulence and survival, especially after macrophagic engulfment. As for the trehalose pathway, some components involved in the glyoxylate cycle are completely absent in mammalian cells—isocitrate lyase (ICL) and malate **s**ynthase (MS) (Chen et al. 2020). Mohangamide A and mohangamide B are efficient inhibitors of ICL in *C. albicans* (Cheah et al. 2014); argentilactone and its semi-synthetic analogs inhibited ICL in *Paracoccidioides*, and some other compounds targeting MS are still under investigation (Prado et al. 2014).

#### Orotomides

With the term orotomides, the microbiological world has identified a completely new class of antifungal drugs. Their target is represented by the dihydroorotate dehydrogenase (DHODH) which is involved in the biosynthesis of pyrimidines (Fig. 3). The depletion of pyrimidines leads to blocks in the DNA/RNA synthesis and in the lipid and carbohydrate precursor productions. Olorofilm F901318 is an excellent inhibitor that can be selective for fungal DHODH even if the enzyme was found also in mammals. The only drawback is the narrow spectrum that includes molds and dimorphic fungi but not yeasts (Singh et al. 2021).

#### The respiratory chain

Among the promising targets, some can be found in the mitochondria (Fig. 3). They are the organelles adhibited to energy production through oxidative phosphorylation and the tricarboxylic acid cycle. Even if some intermediates and enzymes are shared by both fungi and host cells, there are some fungus-specific proteins suitable for new antifungal compound actions (Chatre and Ricchetti 2014). ATI-2307 and ilicicolin H are examples of the application of this approach. The former is an arylamidine able to inhibit both the complex III and IV of the respiratory chain interfering inevitably in the mitochondrial membrane potential (Shibata et al. 2012), while the latter acts on the mitochondrial cytochrome bc_1_ reductase (Singh et al. 2012). Both were tested on *Candida*, *Cryptococcus*, and *Aspergillus* spp., showing great potential, efficacy, and selectivity (Singh et al. 2012). Some other known molecules have been found to have antifungal effects altering the mitochondrial function. For instance, artemisinin seems to lead to mitochondrial dysfunction by affecting the transcription factor pleiotropic drug resistance 1 (PDR1) which regulates the drug efflux pumps and the ergosterol biosynthesis but also downregulates the **N**ADH dehydrogenase Ndi1 of the mitochondrial electron transport chain. Quercetin affects the oxidative stress operating on some electron membrane transport proteins; histatin 5 disrupts the ATP synthesis machinery (e Silva et al. 2020), and VT-1129 and VT-1161 are potent selective inhibitors of the fungal cytochrome P45051 (CYP51). In addition, several antibacterial drugs have demonstrated antifungal properties. Most of them inhibit protein synthesis by binding the ribosome subunits but it seems that the inhibition of particular mitochondrial proteins is extremely deleterious for fungi. Moreover, some antibiotics appear to interfere also with the induction of stress-response mitochondrial chaperones. A few examples are represented by linezolid, azithromycin, and minocycline (Zhang et al. 2021a).

#### The cell wall

Some new strategies, looking at the current antifungal mechanisms, are directed toward the cell wall components or toward the enzymes that produce them (Fig. 1). Nikkomycin Z, for instance, is a competitive inhibitor of the chitin synthase because of the structural similarities with the substrate uridine diphosphate (UDP)-N-acetyl glucosamine (Larwood 2020). It has additive and synergic relationships with azoles and echinocandins in aspergillosis, candidiasis, and coccidioidomycosis (Larwood 2020). Polymyxins like polymyxin B have the same mechanism as nikkomycins and inhibit directly chitin synthase while plagiochin E alters the expression of chitin **s**ynthase genes (CHS). Lectins have a great affinity for polysaccharides like mannose, glucose, and chitin which represent the backbone and the body of the fungal cell wall (Wu et al. 2022). Other examples are represented by the monoclonal antibody IgG2b 2G8 and the humanized versions in different formats, the IgG1 H5K1 (Dia-T51) and the single-chain-fragment variable hscFv (scFv-3T). Their antigens are selectively β-1,3-glucans of the fungal cell wall. The first proved its efficacy in candidiasis, aspergillosis, and cryptococcosis but with the limitation of its murine nature (Di Mambro et al. 2021); while the others have great potential against *Candida* spp., especially when combined with amphotericin B and echinocandins (Di Mambro et al. 2021; Di Mambro et al. 2022; Vanzolini et al. 2023). Rezafungin (SP3025, CD101, or Rezzayo) is a lipopeptide that enters the class of echinocandins, and that in March 2023 got approval in the USA to be used for the treatment of candidemia and invasive candidiasis. It inhibits the β-1,3-glucan synthase enzyme complex, essential for β-1,3-glucans synthesis. Ibrexafungerp (SCY-078 or MK-3118) is a triterpene glycoside and the only FDA-approved antifungal for vulvovaginal candidiasis. As rezafungin, ibrexafungerp inhibits β-1,3-glucan synthase (Sucher et al. 2022; Phillips et al. 2023; Syed 2023). All these ways have the advantage of damaging the wall, proper just of fungi without repercussions for the host.

#### Trivalent cations-related pathways

A different strategy in antimicrobial development addresses iron-related pathways (Fig. 1). This gave the inspiration also for the development of new antifungal drugs. Iron is hijacked by fungal cells and is needed for many processes that confer the typical virulence factors. The main objective is the impairment or the exploitation of the iron assimilation. Different strategies have been adopted. Some antifungal peptides were conjugated to the siderophores in order to be incorporated inside the cells where they are released to carry out their task (Tonziello et al. 2019). Other compounds interfere with the synthesis of siderophores by inhibiting enzymes like nonribosomal peptide synthetases (NRPS), polyketide synthase (PKS), phosphopantetheine transferase (PPtase), and nonribosomal peptide synthetases (NRPS)-independent siderophore **s**ynthetases (NIS). In this context, celastrol inhibits *A. fumigatus* growth by acting on the flavin-dependent monooxygenase siderophore A (SidA) (Sass et al. 2019). Other methods exploit iron chelators or the substitution of iron with gallium (Bastos et al. 2019). Lipocalins, especially ASP-2397, seem to chelate Al^3+^ and Fe^3+^ and to be translocated inside the fungal cell through the siderophore iron transporter 1 (Sit1), preventing the cations hijacking. It showed good selectivity because of the lack of the Sit1 in mammalian cells, and high potency against different fungi (Nakamura et al. 2019). Ciclopirox has a similar mechanism of action sequestering Al^3+^ and Fe^3+^ (Subissi et al. 2010; Oltu et al. 2020a). Salinomycin, deeply studied as an anticancer agent, presents a multimodal mechanism, but the one that appears to be related to the antifungal effect is represented by the complexation of metal cations and their transportation inside the cells by acting as carboxylic polyether ionophore. It is active against some yeasts and filamentous fungi, especially in combination with polymyxin B (Antoszczak and Huczyński 2019).

#### Other targets

Fungal mitosis is another interesting target and, among its inhibitors, the polyketide griseofulvin binds to α and β tubulin, thus inhibiting the formation of the mitotic tube, hence the mitosis and the nuclear acid synthesis (Aris et al. 2022). Haloprogin activity seems related to the inhibition of oxygen uptake and subsequent membrane disruption. It is probably associated with the block of glutathione activity that occurs also for allicin, the molecule of which haloprogin is the active part (Oltu et al. 2020b). With the same rationale, inhibitors of the superoxide dismutases, catalases, and peroxidases are tightly correlated to the remotion of ROS produced by the immune system cells making the fungal cell unable to protect itself (Oltu et al. 2020b). Some cyclooxygenase inhibitors showed antibiofilm activity by inhibiting the prostaglandin E2 biosynthesis (Houšť et al. 2020), and haemofungin has a potent inhibitory activity toward the HemH/ferrochelatase, which is an enzyme that catalyzes the conversion of highly phototoxic porphyrin molecules into haem.

## Concluding remarks

Several natural, semi-synthetic, and synthetic molecules are under investigation as antifungal drugs or as enhancers for commercially available drugs (summarized in Table 1) (e Silva et al. 2020). Concurrently, different repurposing studies are arising to enrich the drug arsenal (Zhang et al. 2021a). No antifungal vaccines have been licensed yet, but research on them and on adjuvants is ongoing. Due to a multiplicity of obstacles, fungal infections keep being a big challenge (Oliveira et al. 2021). Regarding that, metabolomic, proteomic, and lipidomic sciences in combination with computational biology have granted great steps forward in the identification of new targets and compounds in faster times (Amarsaikhan et al. 2017; Brandt et al. 2020; Shahi et al. 2020). We hope that these advancements can also meet facilitated and accelerated paths for entry in the market of new efficient diagnostic and therapeutic tools.

**Table 1:** Summarizing table of the new antifungal options available and under investigation considering their origin, the target/s (or the pathways affected when the target is unknown or undefined yet), and their combinations with other antifungal agents which resulted synergic, additive, or just enhanced

	Name	Origin	Target	Positive combinations with antifungal agents
Cell wall	Nikkomycin Z	Natural	Chitin synthase	Fluconazole, itraconazole, micafungin, caspofungin
	Polymyxins (polymixin B)	Semi-synthetic	Chitin synthase	Amphotericin B, ketoconazole, voriconazole, fluconazole, salinomycin, micafungin
	Plagiochin E	Natural	Alteration of the expression of chitin synthetase genes (CHS)	Fluconazole
	Lectins	Natural	Cell wall polysaccharides (mannose, glucose and chitin)	Fluconazole
	Ibrexafungerp	Semi-synthetic	β-1,3-glucan synthase	Isavuconazole, posaconazole, voriconazole, amphotericin B
	Rezafungin	Semi-synthetic	β-1,3-glucan synthase	
	2G8	Synthetic	β-1,3-glucan	
	H5K1 (Dia-T51)	Synthetic	β-1,3-glucan	Caspofungin, amphotericin B
	hscFv (scFv-3T)	Synthetic	β-1,3-glucan	Caspofungin, amphotericin B
Sphingolipids biosynthesis	BHBM	Synthetic	APL5, COS111, MKK1, and STE2 genes	Tunicamycin, fluconazole, amphotericin B, caspofungin
	D0	Synthetic	APL5, COS111, MKK1, and STE2 genes	Tunicamycin, fluconazole, amphotericin B, caspofungin
	D2	Synthetic	Inhibition of the synthesis of glucosylceramide by regulating vesicle sorting or transport between Golgi apparatus and ER	
	D13	Synthetic	Inhibition of the synthesis of glucosylceramide by regulating vesicle sorting or transport between Golgi apparatus and ER	Itraconazole, voriconazole, fluconazole, caspofungin, amphotericin B
	D17	Synthetic	Inhibition of the synthesis of glucosylceramide by regulating vesicle sorting or transport between Golgi apparatus and ER	
	SB-AF-1002	Synthetic	Inhibition of the synthesis of glucosylceramide by regulating vesicle sorting or transport between Golgi apparatus and ER	Itraconazole
	Aureobasidin A	Natural	Inositol phosphoryl ceramide synthase (IPCS)	Fluconazole
	Myriocin	Natural	Serine palmitoyltransferase (SPT) (hypothesis of Cdr1 as other target)	Fluconazole
	Fumonisins (Fumonisin B1)	Natural	Ceramide synthase (CerS)	
	Australifungin	Natural	Ceramide synthase (CerS)	
	Cerezyme (Imiglucerase)	Synthetic	hydrolyzation of glucosylceramide	Fluconazole
GPI-biosynthesis	Jawsamycin (FR-900848)	Natural	Spt14 subunit of UDP-glycosyltransferase	
	Gepinacin	Synthetic	Inositol acyltransferase (Gwt1)	
	Fosmanogepix/Manogepix	Synthetic	Inositol acyltransferase (Gwt1)	Amphotericin b, anidulafungin
	BIQ	Natural	Inositol acyltransferase (Gwt1)	
	G365	Semi-synthetic	Inositol acyltransferase (Gwt1)	
	G884	Semi-synthetic	Inositol acyltransferase (Gwt1)	
	YW3548/M743	Natural	GPI ethanolamine phosphate transferase 1 (Mcd4)	
	M720	Semi-synthetic	GPI ethanolamine phosphate transferase 1 (Mcd4)	
Hsp90-Calcineurin-PKC axis	Mycograb	Synthetic	Hsp90	Amphotericin b, caspofungin, fluconazole
	Geldanamycin	Natural	Hsp90	Fluconazole, caspofungin, itraconazole, trichostatin A
	17-AAG	Semi-synthetic	Hsp90	Posaconazole, itraconazole, voriconazole, fluconazole, caspofungin
	CMLD013075	Synthetic	Hsp90	Fluconazole, caspofungin, itraconazole, trichostatin A
	Ganetespib	Synthetic	Hsp90	Fluconazole
	Monorden (Radicicol)	Natural	Hsp90	Micafungin, voriconazole
	Tacrolimus (FK506)	Natural	FKBP12	Amphotericin b, caspofungin, fluconazole, itraconazole, voriconazole, posaconazole, isavuconazole, cyclohexamide
	Cyclosporine A	Natural	Cyclophilin-1	Caspofungin, isavuconazole, fluconazole, voriconazole, amphotericin B
	JH-FK-05	Semi-synthetic	FKBP12	Fluconazole
	FK520/Ascomycin	Semi-synthetic	FKBP12	Caspofungin, itraconazole
	L-685,818	Semi-synthetic	FKBP12	Caspofungin, cyclohexamide, itraconazole
	APX879	Semi-synthetic	FKBP12	Fluconazole
	Vasostatin-I	Natural	Inhibition of calmodulin-binding enzymes. Interaction with membrane phospholipids	
	Chromofungin	Synthetic	Inhibition of calmodulin-binding enzymes. Interaction with membrane phospholipids	
	Catestatin	Natural	Inhibition of calmodulin-binding enzymes. Interaction with membrane phospholipids	
	Tamoxifen	Synthetic	Calmodulin	Amphotericin B
	Cercosporamide	Natural	Pkc1	Fluconazole, echinocandin analog ly303366, doxycycline
	Magnolol	Natural	Related to PKC and MAPK-Cek1	Fluconazole, ketoconazole, miconazole, itraconazole, voriconazole
Histone deacetylases cascade	AR-12	Synthetic	Acetyl-CoA synthetase 1 and downregulation of Hsp90 and Hsp27 genes	Fluconazole
	Trichostatin A	Semi-synthetic	Histone deacetylase (HDAC)	Fluconazole, itaconazole, terbenafine
	MGCD290	Synthetic	Hos2 histone deacetylase	Fluconazole, posaconazole, voriconazole, anidulafungin, caspofungin, micafungin
	Nicotinamide	Natural	Hst3 histone deacetylase	Amphotericin B, fluconazole
mTOR	Rapamycin	Natural	FKBP12. Modulates also TORC1, Hsf1 and Hsp90	5-flucytosine, amphotericin B, caspofungin
	Everolimus	Semi-synthetic	FKBP12. Modulates also TORC1	Itraconazole, voriconazole, posaconazole, amphotericin B
Ras pathway	Manumycin A	Natural	Farnesyltransferase	
	Ethylenediamine inhibitor #2	Synthetic	Farnesyltransferase	
	Tipifarnib	Synthetic	Farnesyltransferase	
	Lonafarnib	Synthetic	Farnesyltransferase	Itraconazole, posaconazole, voriconazole
	Farnesyltransferase inhibitor III	Synthetic	Farnesyltransferase	
	Actinoplanic acids (Actinoplanic acid A)	Natural	Farnesyltransferase	Rapamycin
Trehalose pathway	Validamycin A	Natural	Trehalase	Amphotericin B, tebuconazole, propiconazole, prothioconazole
Glyoxylate cycle	Mohangamide A	Natural	Isocitrate lyase (ICL)	
	Mohangamide B	Natural	Isocitrate lyase (ICL)	
	Argentilactone	Natural	Isocitrate lyase (ICL) and UDP-N-acetylglucosamine pyrophosphorylase (UAP)	
Pyrimidine biosynthesis	Orotomides (Olorofim F901318)	Synthetic	Dihydroorotate dehydrogenase (DHODH)	
The respiratory chain	ATI-2307	Synthetic	Mitochondrial respiratory chain complexes III and IV	Fluconazole
	Ilicicolin H	Natural	Mitochondrial cytochrome bc1 reductase	
	Artemisinins (Artemisinin)	Natural	Transcription factor pleiotropic drug resistance 1 (PDR1) and Ndi1	Amphotericin b, fluconazole, miconazole, micafungin
	Quercetin	Natural	Mitochondrial electron transport chain	Fluconazole
	Histatin 5	Natural	ATP synthesis machinery	Amphotericin B
	VT-1129	Synthetic	Cytochrome P45051 (CYP51)	
	VT-1161	Synthetic	Cytochrome P45051 (CYP51)	
Protein synthesis	Linezolid	Synthetic	Inhibition of protein synthesis	Fluconazole, itraconezole, voriconazole, amphotericin B
	Azithromycin	Synthetic	Inhibition of protein synthesis	Terbinafine, amphotericin B, itraconazole, voriconazole, micafungin, caspofungin, anidulafungin, carvacrol, thymol
	Minocycline	Synthetic	Inhibition of protein synthesis	Amphotericin b, fluconazole, itraconazole, voriconazole, posaconazole
Cations and siderophores biosynthesis	Ciclopirox	Synthetic	Chelation of polyvalent metal cations	Clioquinol, terbenafine, ketoconazole, itraconazole
	Salinomycin	Natural	Complexation of the metal cations, the activity depends on the cation transport mediated by the carboxylic polyether ionophore	Polymyxin B
	Celastrol	Natural	Flavin-dependent monooxygenase siderophore A (SidA)	
	VL-2397 (ASP2397)	Natural	Chelation of aluminum, the activity depends on uptake by siderophores transport Sit1	
Others	Haemofungin	Synthetic	HemH/ferrochelatase	Caspofungin
	Griseofulvin	Natural	Binding to α and β tubulin, inhibition of mitosis and nuclear acid synthesis	Micafungin, amorolfine, ketoconazole, fluconazole
	Haloprogin	Semi-synthetic	Inhibition of oxygen uptake and membrane disruption	
